# A Case of Atrioventricular Nodal Re-entrant Rhythm

**DOI:** 10.19102/icrm.2026.17027

**Published:** 2026-02-15

**Authors:** Sudipta Mondal, Nadeem Afroz Muslim, Debargha Dhua

**Affiliations:** 1 Department of Cardiology, The Mission Hospital, Durgapur, India

**Keywords:** Atrioventricular nodal re-entrant rhythm, AVNRR, AVNRT

## Abstract

A 60-year-old male with a history of decompensated chronic liver disease and resolving hepatic encephalopathy was transferred for further evaluation following a witnessed episode of prolonged asystole. The patient’s admission electrocardiogram showed spontaneous transitions between a borderline R–P narrow complex rhythm and sinus rhythm. The rhythm was observed to be easily terminated with ventricular overdrive pacing via the temporary catheter. We questioned what the mechanism of the rhythm may be.

## Case presentation

**Figure 1:**
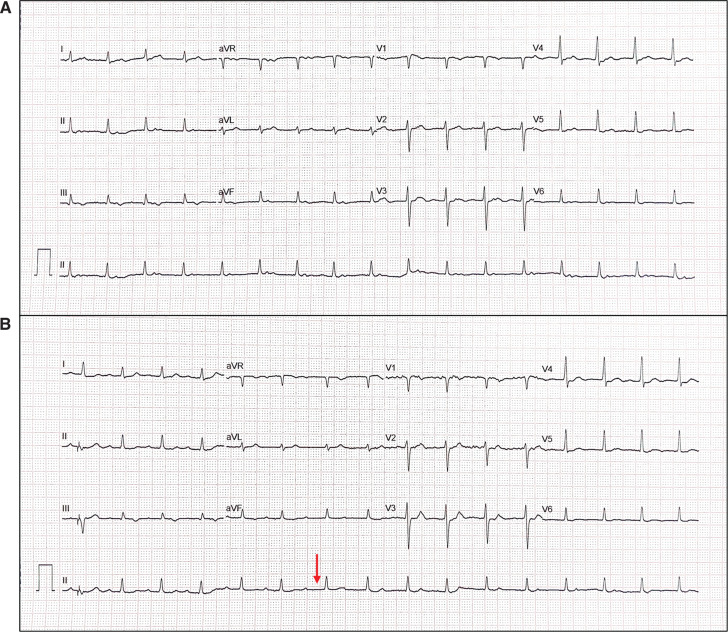
Atrioventricular nodal re-entrant rhythm—diagnosis and mechanisms. **A:** Baseline 12-lead electrocardiogram: This tracing shows a narrow complex rhythm at a rate of 98 bpm. The R–P interval is measured at 160 ms, indicating a long R–P rhythm. **B:** The tracing illustrates the termination and induction of the re–entrant rhythm. The ventricular train by temporary pacing catheter (arrow) successfully terminates the rhythm (termination point not shown). This was reproducible on multiple occasions (termination occurred well before the last paced beat [the first QRS complex in this figure]). This reproducible finding supports the presence of a re–entrant circuit involving the atrioventricular node. The subsequent complexes demonstrate a significant prolongation of the P–R interval (arrow) on the seventh and eighth beats, followed by the re-initiation of the same rhythm. This “A–H jump” phenomenon, characterized by a sudden and significant increase in the A–H interval, is a hallmark of dual atrioventricular nodal physiology, where antegrade conduction switches from a faster pathway to a slower pathway. The spontaneous transition between these pathways highlights the mechanism of the rhythm as an atrioventricular nodal re-entrant rhythm.

A 60-year-old man with a history of decompensated chronic liver disease and resolving hepatic encephalopathy was transferred for further evaluation following a witnessed episode of prolonged asystole. A temporary pacemaker was placed prior to the transfer. A prior Holter monitor demonstrated sick sinus syndrome with a maximum sinus pause of 2.9 s. The patient’s admission electrocardiogram showed spontaneous transitions between a borderline R–P narrow complex rhythm and sinus rhythm **(Figures 1A and 1B)**, both at a rate of 90–100 bpm. The rhythm was observed to be easily terminated with ventricular overdrive pacing (VOP) via the temporary catheter **(Figure 1B)**. We questioned what the mechanism might be.

## Discussion

**Figure 2:**
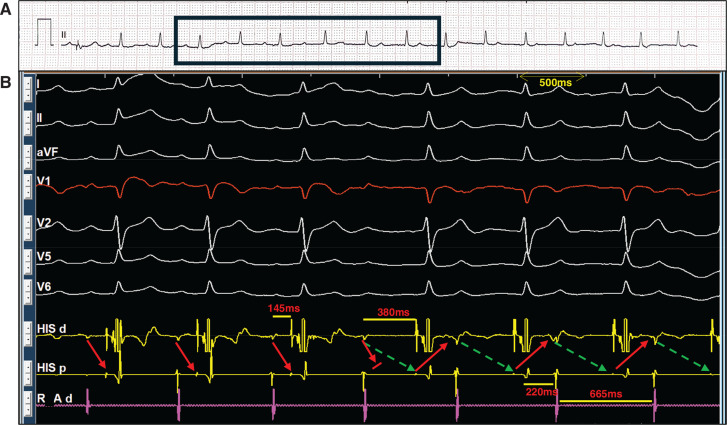
Lead II electrocardiogram **(A)** with corresponding intracardiac electrogram **(B)**, showing the A–H jump (arrow: fast pathway; dashed arrow: slow pathway). This emphasizes the dual atrioventricular nodal physiology. Note: The electrocardiogram and electrogram were taken at different times.

**Figure 3:**
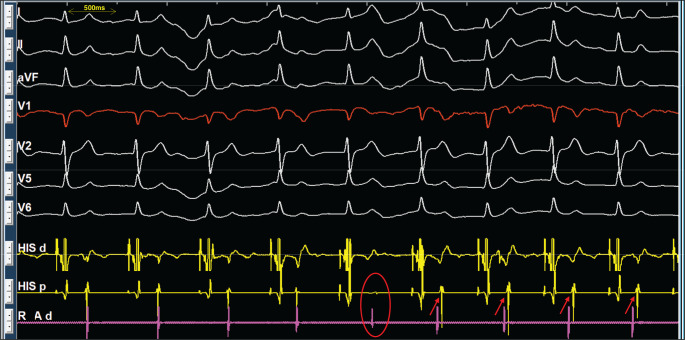
Spontaneous termination of atrioventricular nodal re-entrant rhythm (AVNRR) with a shift to sinus rhythm/right atrial rhythm. The figure shows the termination of an AVNRR. The fifth beat (red circle) marks the spontaneous cessation of the AVNRR and the initiation of a sinus rhythm. Note the change in the atrial activation sequence (arrows). During the AVNRR, the earliest atrial activation is recorded at the His catheter. Following termination, the earliest atrial activation shifts to a location (indicated by the circle), as expected in sinus rhythm/right atrial rhythm. Note: The RA catheter was kept in the low right atrium.

**Figure 4:**
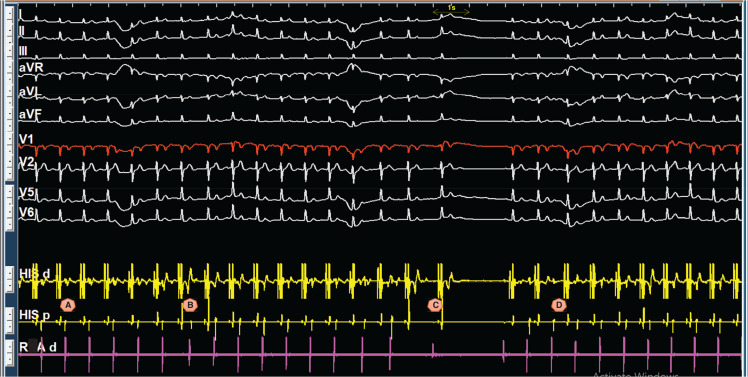
Atypical behavior of the rhythm. **A:** The atrioventricular nodal re-entrant rhythm (AVNRR) is ongoing, with a stable V–A interval, consistent with a re-entrant circuit. **B:** The AVNRR terminates due to a block in the fast pathway, as indicated by a concurrent change in the atrial activation sequence. The earliest atrial activation, which was previously at the His catheter during AVNRR, shifts to a later position during the sinus rhythm/right atrial rhythm that follows. The atrioventricular node then conducts through the slow pathway. **C:** A sudden increase in the A–A interval is noted, reflecting the patient’s documented sinus node dysfunction. This pause facilitates the recovery of the fast pathway, allowing for subsequent conduction through it. **D:** The atrioventricular nodal conduction shifts back from the fast pathway to the slow pathway. The tracing later shows the re-initiation of the re-entrant rhythm (not depicted). The low right atrial catheter position is noted to provide context for the atrial activation sequence.

The presented figures collectively demonstrate a case of atrioventricular (AV) nodal re-entrant rhythm (AVNRR), highlighting its diagnosis and underlying mechanisms. **Figure 1A** shows a long R–P narrow complex rhythm at 90–100 bpm, and its re-entrant nature is confirmed in **Figure 1B** by successful termination with a train of ventricular overdrive and subsequent induction via an “atrial–His (A–H) jump” (a sudden prolongation of the P–R/A–H interval), which, along with **Figure 2**, establishes the presence of dual AV nodal physiology. Intracardiac electrograms in sinus rhythm **(Figure 2B)** showed a prolonged A–H interval, and, in AVNRR, they demonstrated a long ventriculoatrial (VA) interval (220 ms). Slow conduction in the re-entrant circuit contributes to the low heart rate (90 bpm, cycle length, 665 ms) in an ongoing AVNRR. The diagnosis is further supported by **Figure 3**, which illustrates spontaneous termination with a characteristic shift in the earliest atrial activation sequence from the His catheter (during AVNRR) back to the expected location for sinus/right atrial rhythm. Finally, **Figure 4** demonstrates the atypical behavior of the AVNRR, including termination via a fast pathway block, the interplay of sinus node dysfunction (sinus pause facilitating fast pathway recovery), and the subsequent shift in conduction back to the slow pathway, setting the stage for potential re-initiation.

The findings from the electrophysiology study, including the spontaneous termination of the rhythm by atrial premature contractions, VOP, and VA Wenckebach, in conjunction with the prolonged A–H interval, are highly suggestive of an atypical slow-fast AVNRR. In this case, the term “atrioventricular nodal re-entrant rhythm” was adopted to avoid conflict with the fundamental definition of tachycardia. The observed electrophysiological phenomenon is likely a manifestation of significant conduction disease affecting both the fast and slow pathways of the AV node, in addition to the patient’s documented sinus node dysfunction. This complex interplay of pathological states accounts for the spontaneous transitions between rhythms and the atypical nature of the observed rhythm.

Consent for publication was obtained from the patient, in line with Committee on Publication Ethics guidance.

